# A defect in the *NOG* gene increases susceptibility to spontaneous superficial chronic corneal epithelial defects (SCCED) in boxer dogs

**DOI:** 10.1186/s12917-021-02955-1

**Published:** 2021-07-26

**Authors:** Kathryn M. Meurs, Keith Montgomery, Steven G. Friedenberg, Brian Williams, Brian C. Gilger

**Affiliations:** 1 Clinical Sciences, North Carolina State University, 1060 William Moore Drive, Raleigh, North Carolina 27613 USA; 2 Present address: Upstate Veterinary Specialties, Latham, NY USA; 3 Veterinary Clinical Sciences, University of Minnesota, St. Paul, MN USA

**Keywords:** Corneal ulcer, Chronic, Superficial, Recurrent erosion, *NOG*, Boxer

## Abstract

**Background:**

Superficial chronic corneal epithelial defects (SCCEDs) are spontaneous corneal defects in dogs that share many clinical and pathologic characteristics to recurrent corneal erosions (RCE) in humans. Boxer dogs are predisposed to SCCEDs, therefore a search for a genetic defect was performed to explain this susceptibility. DNA was extracted from blood collected from Boxer dogs with and without SCCEDs followed by whole genome sequencing (WGS). RNA sequencing of corneal tissue and immunostaining of corneal sections from affected SCCED Boxer dogs with a deletion in the *NOG* gene and affected non-Boxer dogs without the deletion were performed.

**Results:**

A 30 base pair deletion at a splice site in Noggin (*NOG*) (Chr 9:31453999) was identified by WGS and was significantly associated (*P* < 0.0001) with Boxer SCCEDs compared to unaffected non-Boxer dogs. NOG, BMP4, MMP13, and NCAM1 all had significant fold reductions in expression and SHH was significantly increased in Boxers with the *NOG* deletion as identified by RNA-Seq. Corneal IHC from *NOG* deletion dogs with SCCEDs had lower NOG and significantly higher scores of BMP2.

**Conclusions:**

Many Boxer dogs with SCCED have a genetic defect in *NOG*. *NOG* is a constitutive protein in the cornea which is a potent inhibitor of BMP, which likely regulate limbal epithelial progenitor cells (LEPC). Dysregulation of LEPC may play a role in the pathogenesis of RCE.

**Supplementary Information:**

The online version contains supplementary material available at 10.1186/s12917-021-02955-1.

## Background

Superficial chronic corneal epithelial defects (SCCEDs), also known as indolent corneal ulcers, are spontaneous corneal defects in dogs that share many clinical, pathophysiologic, and pathologic characteristics with recurrent corneal erosions (RCE) in humans [1–3]. These similarities include a spontaneous and recurrent nature, associated ocular discomfort, epithelial loss with a non-adherent epithelial border, a paracentral lesion location, and a lack of infectious organisms [1–6]. Morphologic similarities also include a reduced adhesion of the corneal epithelium to the underlying extracellular matrix, a deficient epithelial basement membrane, absence and abnormality of basal epithelial cell hemidesmosomes, and a loss of anchoring fibrils from the basal epithelium to the stroma [2, 3, 6]. Furthermore, a reduction of corneal innervation was identified in dogs with SCCEDs, thus resembling neurotrophic corneal ulcers in humans [1]. RCEs and SCCEDs may be primarily the result of a defect in the epithelial basement membrane or poor epithelial healing from complete or partial limbal stem cell or limbal progenitor cell deficiencies [7–9]. RCEs may also be secondary to corneal degenerative diseases, dry eye, eyelid abnormalities, previous corneal surgery, and systemic diseases, such as diabetes, among other causes [3, 6].

Treatment of SCCEDs or RCEs remains challenging and therapies for these chronic, painful conditions have not changed substantially in the past several decades. Conservative treatments, such as epithelial debridement, topical antibiotics, and artificial tears are commonly used but rarely effective. Unresponsive patients may benefit from use of therapeutic bandage contact lenses, amnion membrane grafts, matrix metalloproteinase (MMP) inhibitors (topical or systemic), and/or topical serum eye drops. Diamond burr debridement, anterior stromal puncture, alcohol delamination, or superficial keratectomy are used in resistant cases, especially when the patient has suspected epithelial basement membrane dystrophies [3–6, 10–15]. Effective treatments that target the underlying cause of both SCCEDs and RCEs remain elusive.

Boxer dogs are highly predisposed to development of SCCEDs, however, the underlying cause is unknown [4, 16–18]. To determine if there is an underlying genetic abnormality to describe this predisposition, and to investigate the underlying pathogenesis of SCCEDs and RCEs (i.e., using the Boxer dog as a naturally-occurring model for RCEs), a search for a genetic defect was performed in Boxer dogs. Using whole genome sequencing (WGS), we identified a 30-base-pair deletion at a splice site in the noggin gene (*NOG*), which was significantly associated (*P* < 0.0001) with Boxer dogs with SCCEDs compared to unaffected non-Boxer dogs. Furthermore, RNA sequencing of corneal samples and immunostaining of keratectomy specimens from SCCED Boxer dogs deficient in *NOG* revealed reduced expression of *NOG* and alteration of RNA expression of several factors in the BMP signaling pathway compared to non-Boxer SCCED dogs that were wild type (WT) for the *NOG* deletion. These results suggest that *NOG* deficiency and effect on the BMP signaling pathway may impact limbal cell progenitor cells and play a role in SCCEDs and RCEs.

## Methods

Use of animals in this study adhered to the Association for Research in Vision and Ophthalmology statement on the use of animals in ophthalmic and vision research. The animal use protocol was approved and monitored by the North Carolina State University Institutional Animal Care and Use Committee (IACUC) (Protocol # 18–164-0). The North Carolina State University Veterinary Hospital Board approved the protocol for evaluation of clinical patients in this study. Owners of dogs signed and provided informed consent for all sample collections and data collection. The study was carried out in compliance with the ARRIVE guidelines.

### Animal selection/phenotyping

Whole blood samples were collected from Boxer dogs either with, or having a documented history (clinical cases of the NC State Veterinary Teaching Hospital Ophthalmology service) of having SCCED, and Boxer dogs over 10 years of age (clinical cases of the NC State Veterinary Teaching Hospital Cardiology service). with no known history of a SCCED and no evidence of previous corneal disease as examined by an NC State University veterinary ophthalmologist. All blood was collected in EDTA tubes and DNA was extracted using the standard protocol of the DNeasy Blood and Tissue Kit (Qiagen, Germantown, MD).

### Whole genome sequencing

Samples from 8 SCCED Boxer dogs were submitted for library preparation and whole genome sequencing, using a 150 base pair (bp) paired-end read configuration on an Illumina HiSeq 4000 high-throughput sequencing system (Genewiz LLC, South Plainfield, NJ).

Variant calling from next-generation sequencing data was performed using a standardized bioinformatics pipeline for all samples, as described previously [19]. Sequence reads were trimmed using Trimmomatic 0.32 to a minimum phred-scaled base quality score of 30 at the start and end of each read, with a minimum read length of 70 bp, and aligned to the canFam3 reference sequence using BWA 0.7.13 [20–22]. Aligned reads were prepared for analysis using Picard Tools 2.8 (http://broadinstitute.github.io/picard) and GATK 3.7 following best practices for base quality score recalibration and indel realignment (Broad Institute, Cambridge, MA, USA) [23, 24]. Variant calls were made using GATK’s HaplotypeCaller walker, and variant quality score recalibration (VQSR) was performed using sites from dbSNP 146 and the Illumina 174 K CanineHD BeadChip as training resources. A VQSR tranche sensitivity cutoff of 99.9% to SNPs and 99% to indels was used for downstream analyses; genotype calls with a phred-scaled quality score < 20 were flagged but not removed from the variant callset.

Variants (heterozygous or homozygous) present in at least 7/8 affected Boxers were identified. The resulting variants were then filtered against a previously established database of variants from 84 non-Boxer dogs of 17 different dog breeds without a known risk for development of SCCED (https://cidd.discoveryspace.ca/). (Supplemental data Table 1) Any variants with a minor allele frequency greater than 1% in the non-Boxer dogs were removed. The remaining variants were categorized by Variant Effect Predictor 91 (https://useast.ensembl.org/info/docs/tools/vep/index.html) and prioritized by their functional impact (e.g., stop codon, frameshift, change in amino acid, etc.). They were manually curated for potential role in corneal disease and corneal wound healing, such as bone morphogenetic proteins (BMPs), transforming growth factor beta superfamily, noggin, etc. Missense variants were evaluated for genomic functional significance with Polyphen (http://genetics.bwh.harvard.edu/pph2/), SIFT (http://sift.jcvi.org/) and Provean (http://provean.jcvi.org/index.php). In Silico Splice Site Analysis was performed with Human Slice Site Finder (http://www.umd.be/HSF/) to predict the potential impact of splice site changes.

Variants involved with genes associated with a potential role in corneal disease and/or corneal wound healing were prioritized based on the variant’s impact on the gene (deleterious missense, stop/start gained or lost, inframe deletion, frameshift) and were pursued with Sanger Sequencing in the additional affected (62) Boxer dogs and apparently unaffected (25) Boxer dogs and tested for allelic association with SCCED using a Fisher’s exact test. A *p*-value of < 0.05 was considered significant.

Variants of strongest interest were subsequently filtered against a larger established canine database of 391 non-Boxer dogs of 53 different breeds to assess variant prevalence in the canine population.

### RNA library preparation and sequencing

To assess RNA expression of genes in corneal tissue associated with SCCED, total RNA was extracted from corneal tissue gently debrided from four SCCED Boxer dogs with the *NOG* deletion and three affected non-Boxer dogs without the *NOG* deletion (Boston Terrier (2), Pomeranian) as has been described previously [25]. Using the Qiagen RNeasy Plus Universal mini kit (Qiagen, Germantown, MD) RNA was quantified using Qubit 2.0 Fluorometer (Life Technologies, Carlsbad, CA) and RNA integrity was checked with Agilent TapeStation (Agilent Technologies, Palo Alto, CA). RNA library preparation, sequencing, and initial bioinformatics analysis was conducted at GENEWIZ, LLC (South Plainfield, NJ). The RNA sequencing library preparation was performed with the NEBNext Ultra RNA Library Prep Kit (NEB, Ipswich, MA, USA) for Illumina (San Diego, CA) followed by manufacturer’s instructions. The sequencing library was validated on an Agilent TapeStation (Agilent Technologies, Palo Alto, CA, USA), and quantified with a Qubit 2.0 Fluorometer (Invitrogen, Carlsbad, CA**)** as well as by quantitative PCR (KAPA Biosystems, Wilmington, MA, USA). The sequencing libraries were clustered on one lane of a flowcell. After clustering, the flowcell was loaded on the Illumina HiSeq 4000 high-throughput sequencing system instrument according to manufacturer’s instructions. The samples were sequenced using a 2 × 150 Paired End (PE) configuration. Image analysis and base calling were conducted by the HiSeq Control Software. Raw sequence data (.bcl files) generated from Illumina HiSeq was converted into fastq files and de-multiplexed using Illumina’s bcl2fastq 2.17 software. One mis-match was allowed for index sequence identification.

### RNA-Seq analysis

The datasets and transcript counts were estimated using Salmon (version 0.11.2) in quasi-mapping mode against dog reference genome CanFam3.1 with default settings [26]. Salmon estimated counters were summarized to gene level using the tximport package in R (v 3.4.4) for use with DESeq2 [27]. DESeq2 was used for differential expression testing. Differential gene expression analysis was performed by comparing samples from Boxer SCCED cases to non-Boxer SCCED cases. A cutoff of |log_2_ fold change| > 1.5 was considered significant [28].

Ingenuity Pathway Analysis (IPA) (Qiagen, Redwood CA) was used to evaluate key biologic pathways involving NOG to identify genes that could be impacted by altered NOG for further assessment with RNA Seq and immunohistochemical analysis.

### Immunohistochemical analysis of corneal specimens from SCCED dogs

Keratectomies were performed to treat the SCCED in five Boxer dogs with the *NOG* deletion and three affected non-Boxer dogs without the *NOG* deletion. Corneal keratectomy specimens were preserved for future analysis and were fixed in 4% buffered paraformaldehyde overnight at 4 °C and then transferred to 70% ethanol before being embedded in paraffin. Tissues were sectioned at 5 μm and stained with hematoxylin and eosin. Immunofluorescence was performed following a previously described method with immunohistochemistry antibodies selected for the gene of interest and genes in the biologic pathway predicted to be impacted by altered gene function [29]. In short, sections were deparaffinized by incubating the slides two times in xylene for 10 min (min) each, followed by immersing the slides sequentially in two rounds of 100% (3 min each), 95% (1 min), and 80% (1 min) ethanol solutions, and finally in water for 5 min. Antigen retrieval procedure was performed by heating the slides to 95 °C in citrate-based (pH 6.0) antigen unmasking buffer (Vector Laboratories) before staining. Non-specific staining was blocked by using PBS containing 10% of normal goat serum, 0.025% Triton X-100 plus 1% BSA before overnight incubation with the primary antibody. The primary antibodies included rabbit polyclonal NOG 1:100 (Abcam ab16054); rabbit polyclonal BMP2 (Novus NBP1-19751SS), rabbit polyclonal BMP4 (Abnova PAB3673). Negative controls were performed as described above but without use of a primary antibody. After the staining, slides were mounted and counter stained with ProLong™ Diamond Antifade Mountant with DAPI ﻿(p36971, Invitrogen) and observed by Olympus IX83 Fluorescence Microscope (Olympus, Tokyo, Japan) or Zeiss LSM 780 inverted confocal microscope. Intensity and distribution of IHC staining was scored by two, blinded, experienced examiners using the following scoring scheme; 0 = no staining; 1 = slight, focal corneal staining; 2 = slight, diffuse staining; 3 = moderate intensity, focal to diffuse; 4 = high intensity, diffuse staining. Results of the two observers were averaged to provide a final score for each specimen. Pairwise Wilcoxon (Mann-Whitney tests) were performed to evaluate for group differences in IHC scores using JMP version 14.0 (SAS Institute Cary, NC).

## Results

### Animal selection/phenotyping

Seventy Boxer dogs either with, or having a documented history of SCCED and twenty-five Boxer dogs at least 10 years of age with no known history of SCCED were identified.

### Whole genome sequencing

After filtering, 5013 variants were identified in at least 7 of 8 affected Boxers and in less than 1% of the 84 non-Boxer dogs from 17 different dog breeds. After filtering for genes identified as those likely to have a possible role in corneal disease and/or corneal wound healing, eight variants remained in five genes, *KIF5C, NOTCH1, EMCS, Serpine2* and *NOG*. (Table 1) These variants were all pursued by Sanger sequencing. The variants in *KIF5C* were all predicted to be low impact (intronic, non-splice site SNPs) but were pursued anyway because of the apparent involvement of the gene in the cornea. All remaining variants were in genes without a clear likely role in corneal disease and/or corneal wound healing and were SNPs or small indels located in a 3′ or 5′ untranslated, upstream, downstream or intronic region. These were not evaluated further.

**Table 1:** Variants identified in Boxers dogs with SCCED in genes likely to have a possible role in corneal disease and corneal wound healing

Chromosomal location	Gene	Reference allele	Variant allele	Gene location
19: 50605253	KIF5C	C	A	Intronic
19: 50602128	KIF5C	A	T	Intronic
19: 50543167	KIF5C	T	A	Intronic
19: 50546726	KIF5C	C	A	Intronic
32: 22284093	EMCS	A	G	Missense
37: 29701267	Serpine 2	A	G	Splice Site
9: 49015931	NOTCH1	G	A	Missense
9: 31453999	NOG	gtgtgtgtgtgtgt gtgtgtgtgtgtgtga		Deletion

These eight variants were further evaluated by Sanger Sequencing. The variants in *KIF5C, NOTCH1* and *EMCS* were inconsistently found in the affected dog population and did not have a statistically significant association with SCCED. In contrast, the variants in *Serpine2* and *NOG* were found in all of the SCCED Boxer dogs but also were found in 23 of 25, and 24 of 25 of the Boxers without SCCED, respectively.

When evaluated against the larger canine database of 391 non-Boxer dogs of 53 different breeds, the *SERPINE2* variant was also found in 22 dogs of 11 breeds (5% of the overall dog population) while the *NOG* variant was found in only 4 dogs of one additional breed (Yorkshire Terrier) (less than 1% of the overall dog population).

The *NOG* variant was a 30 base pair deletion at a splice site (Chromosome 9:31,453,999-31,454,029, ENSCAFT00000047287.1:c.46 + 13_46 + 42delGTGTGTGTGTGTGTGTGAGTGTGTGTGTGT). In silico splice site analysis predicted the *NOG* variant to disrupt a splice site as well as an enhancer motif site.

### RNA-Seq

With a cutoff of |log_2_ fold change| > 1.5 considered significant, *Serpine2,* was not differentially expressed (0.015 log_2_ fold) between the SCCED groups while.

**Fig. 1:**
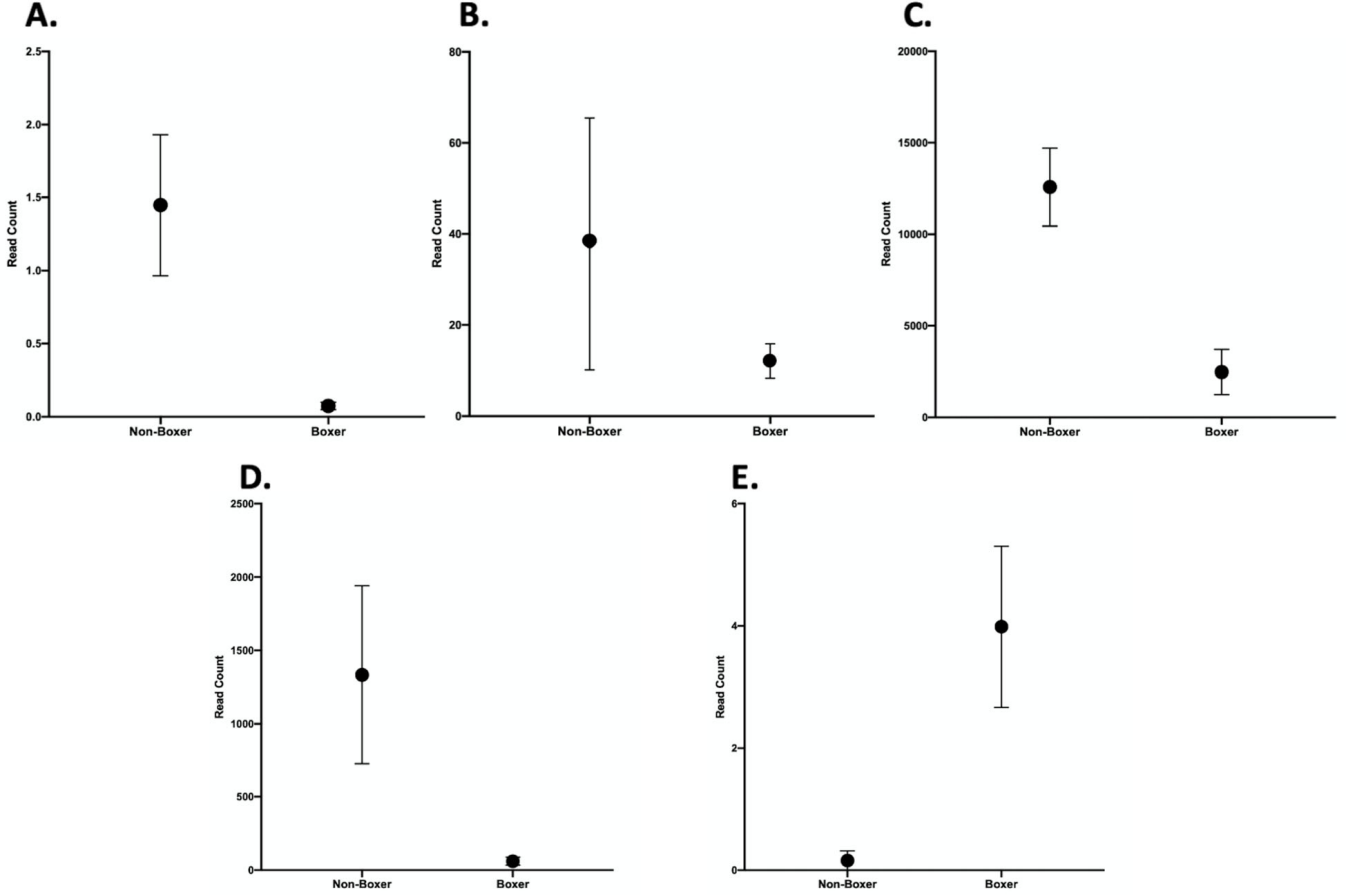
RNA-Seq of corneal tissue from 4 SCCED Boxer dogs and 3 SSCED non-Boxer dogs demonstrating differential expression of *NOG* and four genes within the BMP Signaling Pathway *BMP4, MMP13, NCAM1* (all reduced) and *SHH* (increased). All were found to be differentially expressed by comparing samples from Boxer SCCED cases to non-Boxer SCCED cases. A cutoff of |log_2_ fold change| > 1.5 was considered significant. Mean and standard error is shown. A.NOG B. BMP4 C. MMP13 D. NCAM1 E. SHH

*NOG* expression was reduced (2.8 log_2_ fold). (Supplemental Data Table 2) Four genes within the BMP Signaling Pathway which were expressed in the cornea were found to be differentially expressed including BMP4, MMP13, NCAM1 (all reduced) and SHH (increased) (Table 2) (Fig. 1A-E).

**Table 2:** Differential expression of NOG and genes that NOG regulates in the BMP Signaling Pathway from SCCED Boxer dogs with *NOG* deletion compared to SCCED non-Boxer without the *NOG* deletion

Gene	Fold Change^a^
NOG	−2.8
Alp	Not expressed
BMP	Not expressed
BMP2	NS
BMP4	−1.6
FGF8	Not expressed
LEF1	NS
MMP13	−2.3
NCAM1	−4.4
RUNX2	Not expressed
SHH	3.9
SMAD1	Not expressed
VEGFA	NS

^a^Not expressed indicates that measurable amounts were not detected in the sample, NS indicates that the fold change was less than 1.5

### Corneal immunohistochemistry

**Fig. 2:**
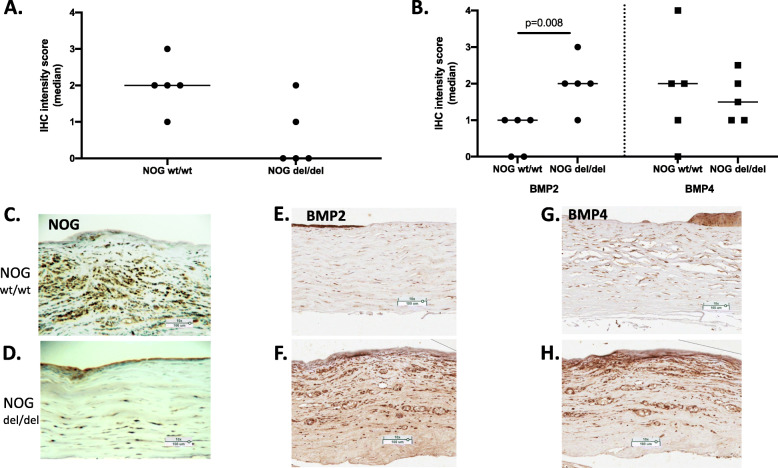
Immunohistochemistry (IHC) Scores of Keratectomy Specimens of Dogs with Chronic non-healing Corneal ulcers (Scatterplot, median). **A** NOG staining. Samples from NOG del/del dogs had a lower mean NOG IHC score than samples from NOG wt/wt, but the difference was not significant (*p* = 0.0625). **B** BMP2, and 4 IHC scores of samples from NOG del/del and NOG wt/wt dogs. Keratectomy specimens from NOG del/del dogs had significantly higher BMP2 (*p* = 0.008) than samples from NOG wt/wt dogs (Man Whitney U Test). Representative IHC images of keratectomy samples from NOG wt/wt and NOG del/del dogs. C. NOG score 3; D. NOG score 1; E. BMP2 score 1; F. BMP2 score 3; G.BMP4 score 1; H. BMP 4 score 3. 10X Magnification

Samples from Boxer SCCED dogs with the *NOG* deletion had a significantly reduced IHC score (*p* = 0.03) compared to mean IHC scores from corneal samples from non-Boxer SCCED dogs without the *NOG* deletion (Fig. 2). Keratectomy specimens from Boxer SCCED dogs had significantly higher BMP2 (*p* = 0.01) scores than corneal samples from non-Boxer SCCED dogs (Wilcoxon Test) although it was not found to be significantly different by RNA Seq. (Supplemental data Table 2) BMP4 appeared to be reduced but the differences were not significant by IHC (Fig. 2 A-H).

## Discussion

Our data have demonstrated that many Boxer dogs with superficial nonhealing corneal ulcers have a genetic defect in the *NOG* gene (a 30 base pair deletion at a splice site in *NOG* [Chromosome 9:31,453,999-31,454,029]), which encodes the protein noggin, a constitutively-secreted, 46 kDa, disulfide-linked homodimer consisting of two, 206 amino acid polypeptide chains [30]. Not only was the genetic *NOG* defect highly associated with SCCED Boxer dogs, but these *NOG* deficient dogs also had greater than two fold reduction of corneal epithelial noggin RNA signal and significantly reduced noggin staining in the stroma of keratectomy samples compared to *NOG*^+^ SCCED dogs. Noggin is involved in the bone morphogenetic protein (BMP) pathway where it serves as a potent inhibitor of BMP of the transforming growth factor-β superfamily. Noggin binds to BMPs receptor complex (BMPR-I) preventing its activation [30, 31]. In the Boxer dogs with SCCED evaluated here the reduction of Noggin was identified, as well as alterations in expression of several other key factors in the BMP Signaling pathway including NCAM1, MMP13 and SHH (Table 2). Noggin regulation should increase expression of NCAM1 and MMP13, and decrease expression of SHH. In the dogs reported here, we identified decreased NCAM1 and MMP13 and increased SHH as would be expected with reduced levels of NOG (Table 3). Specifically, noggin inhibits BMP2, 4, 5, 7, 13 and 14, but leaves BMP3, 6, 9, 10 and 15 signaling unaffected [30]. Both BMP2 and BMP4 are also expected to be inhibited by NOG. Here we found BMP2 to have a measurable increase according to the corneal stroma IHC analysis, although not in the epithelium by RNA, as would be expected with a reduction in NOG. BMP4, was decreased at the RNA level. BMP4 is both negatively regulated by NOG, as well as having a role in regulating NOG and its response to reduced NOG may be more complex.

**Table 3:** Immunohistochemistry scoring of NOG and genes that NOG regulates in the BMP Signaling Pathway from SCCED Boxer dogs with *NOG* deletion compared to SCCED non-Boxer without the *NOG* deletion

	***NOG***^**−**^ IHC score (Mean ± SD)	***NOG*** WT score (Mean ± SD)	***P*** value
NOG	0.6 ± 0.9	2.0 ± 0.7	0.03
BMP4	1.6 ± 0.7	2.0 ± 1.6	0.62
BMP2	2.0 ± 0.7	0.6 ± 0.5	0.01

This study demonstrated a significant association between a genetic *NOG* defect, decreased tissue noggin, alterations in the BMP signaling pathway and delayed corneal epithelial wound healing in dogs diagnosed with SCCED. Several studies have also observed reduced keratinocyte proliferation and delayed dermal wound healing associated with reduced noggin and increased BMP activity [30, 32]. BMPs, BMP receptors, and noggin have been shown to be expressed in human corneal epithelial cells and keratocytes, and they likely regulate limbal stem cells and corneal epithelial wound healing [33, 34]. Limbal stem cells reside in a cellular complex called the stem cell niche [35], consisting of the limbal epithelial progenitor cells (LEPC) and limbal niche cells (LNC), the latter of which controls self-renewal and fate decisions of LEPCs partially through production of noggin [34]. Noggin suppresses BMPs in LEPCs through suppression of the SMAD signaling pathway and activates canonical Wnt signaling which is correlated with clonal growth of LEPCs [34]. Therefore, Noggin deficiency would allow BMP to bind to BMPR-I, initiate SMAD signaling, reduce Wnt signaling, and suppress LEPC growth, all of which may contribute to development of SCCEDs.

Although BMPs and Noggin effect on corneal wound healing has not been well described, BMPs and noggin have been shown to have an effect on dermal wound repair and healing. BMP2 and BMP4 have been demonstrated to reduce keratinocyte proliferation [36, 37] and transgenic mice overexpressing SMAD1 (part of the BMP signaling cascade) have delayed dermal wound healing [32]. Furthermore, exogenous topical administration of BMP4 and 7 to a human ex vivo dermal wound healing model impaired epithelial wound closure [32]. Overall, the effect of BMPs is to suppress keratinocyte proliferation and increase apoptosis in the dermal wound epithelium, thus negatively regulate keratinocyte proliferation and migration during wound healing [32]. Alternatively, noggin has been shown to improve dermal wound healing. Transgenic mice that overexpress noggin have accelerated dermal wound healing, increased keratinocyte proliferation, and increased wound vasculature compared to wild type mice [32]. Exogenous topical treatment with noggin to a human ex vivo dermal wound healing model resulted in accelerated epithelial wound closure [32]. Therefore, inhibiting BMPs or providing exogenous noggin may improve dermal and corneal epithelialization; and provide a potential therapeutic for SCCED and chronic recurrent erosions.

*NOG* mutations have been reported in humans as an autosomal dominant disease with variable phenotype, commonly referred to as symphalangism spectrum disorder (*NOG*-SSD), involving 5 main autosomal dominant syndromes: (1) proximal symphalangism; (2) multiple synostoses syndrome 1; (3) stapes ankylosis with broad thumbs and toes; (4) tarsal-carpal coalition syndrome; and (5) brachydactyly type B2 [38–40]. Other than hyperopia and strabismus, which is common, ocular abnormalities have not been reported in these patients [40]. Boxer dogs with SCCEDs have not been reported to have orthopedic or joint disorders, nor did the dogs with the *NOG* deletion included in this study.

In the dogs evaluated here, Boxer dogs without a known SCCED history also commonly had the *NOG* deletion. If noggin deficiency is associated with suppression of LEPC growth one might hypothesize that many Boxers will have a normal phenotype until they suffer a corneal abrasion and at that point the *NOG* deletion and its impact on the BMP signaling pathway could prevent the LEPC growth and normal healing that would occur in a dog without this deletion. Only at that time would the SCCED phenotype become apparent. Additionally, since the deletion is not one that would shorten life span or impact successful reproduction it would not have been naturally selected against and could be quite widespread within the breed. However, the pathogenesis of SCCED in dogs has been reported to a reduced adhesion of the corneal epithelium to the underlying corneal extracellular matrix and not a result of reduced corneal epithelial growth [1, 2, 17]. It is possible that Noggin deficiency may negatively regulate stromal keratinocytes, as seen in dermal wound healing [32], leading to changes in extracellular matricies or epithelial basement membrane in the cornea. These changes may then lead to reduced adhesion of the corneal epithelium to the underlying extracellular matrix and corneal stroma in SCCED [1, 2, 17]. Further study of the role of Noggin and the effects of its deficiency o on corneal wound healing and on the development of SCCED in dogs is needed.

In this study, using whole genome sequencing (WGS), we identified a 30 base pair deletion at a splice site in the noggin gene (*NOG*) which was significantly associated with Boxer dogs with SCCEDs compared to non-Boxer dogs. Furthermore, RNA sequencing of corneal samples and immunostaining of keratectomy specimens from SCCED Boxer dogs deficient in *NOG* revealed reduced RNA signal and expression of *NOG*, respectively, and altered expression of BMP signaling pathway factors compared to WT dogs. A limitation of this study is that we compared corneal tissue from Boxer dogs with SCCEDs to non-Boxer dog with SCCEDs, rather than comparing it to corneal tissue from unaffected control dogs. Since SCCEDs are likely to have RNA and protein expression alterations that occur as a consequence of the corneal ulcer, comparison to unaffected corneal tissue could have emphasized changes that occur due to SCCED rather than the underlying cause. Finally, additional analysis of the NOG RNA for alignment and transcript analysis would have been useful but unfortunately the quality and quantity of NOG RNA from debrided cells was quite limited and prevented additional transcript analysis.

## Conclusions

Many Boxer dogs with SCCED have a genetic defect in *NOG*. *NOG* is a constitutive protein in the cornea which is a potent inhibitor of BMP, which likely regulate limbal epithelial progenitor cells (LEPC). Dysregulation of LEPC may play a role in the pathogenesis of RCE. These results suggest that *NOG* deficiency play a role in SCCEDs and RCEs. Further study of this defect in the pathogenesis of SCCED and RCE appears warranted.

## Supplementary Information

**Additional file 1.**

## Data Availability

The datasets used and/or analyzed during the current study are available from the corresponding author on reasonable request.

## Abbreviations

BMP: Bone morphogenic protein
Chr: Chromosome
IACUC: Institutional animal care and use committee
ICH: Immunohistochemistry
LEPC: Limbal epithelial progenitor cells
NOG: Noggin
RCE: Recurrent corneal erosions
SCCED: Superficial chronic corneal epithelial defects
WGS: Whole genome sequencing
