# Supplementary material for: A defect in the NOG gene increases susceptibility to spontaneous superficial chronic corneal epithelial defects (SCCED) in boxer dogs
Source: BMC Vet Res. 2021 Jul 26;17:254. doi: 10.1186/s12917-021-02955-1 (PMC8314488; doi:10.1186/s12917-021-02955-1)
Supplement: Supplementary file 1 — Additional file 1. [file 12917_2021_2955_MOESM1_ESM.pdf]

A defect in the *NOG* gene increases susceptibility to spontaneous superficial chronic corneal epithelial defects (SCCED) in Boxer dogs

Kathryn M. Meurs[1], Keith Montgomery[1*], Steven G. Friedenberg[2], Brian Williams[1], Brian C. Gilger[1]

[1]Clinical Sciences, North Carolina State University, Raleigh, NC, USA

[2]Veterinary Clinical Sciences, University of Minnesota, St. Paul, MN, USA

*Current location, Upstate Veterinary Specialties, Latham, NY, USA

Supplemental data

Supplemental Table 1. Representative dog breeds without a known risk for SCCED used filtering variants

Dog Breed
Cavalier King Charles Spaniels
Collie
Dachshund
Doberman Pinscher
German Shepherd
Golden Retriever
Great Dane
Irish Setter
Miniature Poodle
Rhodesian Ridgeback
Scottish Deerhound
Scottish Terrier
Shetland Sheepdog
Standard Poodle
Toy Poodle
West Highland White Terrier
Yorkshire Terrier

Supplemental Table 2. RNA Seq expression of genes that regulate NOG

Gene	*Fold Change
BMP2	NS
BMP4	-1.6
BMP6	NS
FGFR2	NS
FGFR3	NS
FGF18	NS
FGF8	Not expressed
FXR2	NS
GDF2	NS
GSK2816126	Not expressed
HOXA13	-1.4
NEUROG1	Not expressed
RASSF1	NS
SOX2	NS
TGFB1	NS

*Not expressed indicates that measurable amounts were not detected in the sample, NS indicates that the fold change was not significant
